# Exploring the role narrative free-text plays in discrepancies between physician coding and the InterVA regarding determination of malaria as cause of death, in a malaria holo-endemic region

**DOI:** 10.1186/1475-2875-11-51

**Published:** 2012-02-21

**Authors:** Johanna C Rankin, Eva Lorenz, Florian Neuhann, Maurice Yé, Ali Sié, Heiko Becher, Heribert Ramroth

**Affiliations:** 1 Institute of Public Health, University Hospital of Heidelberg, Heidelberg, Germany; 2 Centre de Recherche en Santé de Nouna, Nouna, Burkina Faso

**Keywords:** Verbal autopsy, Malaria, Free-text, INDEPTH, Cause of death, Burkina Faso, Bayesian InterVA model

## Abstract

**Background:**

In countries where tracking mortality and clinical cause of death are not routinely undertaken, gathering verbal autopsies (VA) is the principal method of estimating cause of death. The most common method for determining probable cause of death from the VA interview is Physician-Certified Verbal Autopsy (PCVA). A recent alternative method to interpret Verbal Autopsy (InterVA) is a computer model using a Bayesian approach to derive posterior probabilities for causes of death, given an *a priori *distribution at population level and a set of interview-based indicators. The model uses the same input information as PCVA, with the exception of narrative text information, which physicians can consult but which were not inputted into the model. Comparing the results of physician coding with the model, large differences could be due to difficulties in diagnosing malaria, especially in holo-endemic regions. Thus, the aim of the study was to explore whether physicians' access to electronically unavailable narrative text helps to explain the large discrepancy in malaria cause-specific mortality fractions (CSMFs) in physician coding versus the model.

**Methods:**

Free-texts of electronically available records (N = 5,649) were summarised and incorporated into the InterVA version 3 (InterVA-3) for three sub-groups: (i) a 10%-representative subsample (N = 493) (ii) records diagnosed as malaria by physicians and not by the model (N = 1035), and (iii) records diagnosed by the model as malaria, but not by physicians (N = 332). CSMF results before and after free-text incorporation were compared.

**Results:**

There were changes of between 5.5-10.2% between models before and after free-text incorporation. No impact on malaria CSMFs was seen in the representative sub-sample, but the proportion of malaria as cause of death increased in the physician sub-sample (2.7%) and saw a large decrease in the InterVA subsample (9.9%). Information on 13/106 indicators appeared at least once in the free-texts that had not been matched to any item in the structured, electronically available portion of the Nouna questionnaire.

**Discussion:**

Free-texts are helpful in gathering information not adequately captured in VA questionnaires, though access to free-text does not explain differences in physician and model determination of malaria as cause of death.

## Background

In countries where the lack of health surveillance infrastructure renders tracking mortality and clinical cause of death very difficult, gathering verbal autopsies from next of kin is the principal method of estimating cause of death, despite known limitations [1-5]. Trained field workers conduct questionnaire-based face-to-face interviews with the deceased's nearest contact at time of death about signs, symptoms and circumstances of death, within several months following a person's death. Typically, each questionnaire is then independently reviewed by physicians, each giving their diagnosis. Up to three physicians take part in this process, with a third physician required in as many as 50% of questionnaires due to a lack of consensus between the first two [6], a method allowing for possible bias and human error. In the past decade, a mathematical model called InterVA (interpretation of verbal autopsies) has begun to be used in developing countries, most notably in the INDEPTH network, estimating cause of death based on Bayesian probability models [7].

Burkina Faso is one of the poorest countries in the world, 46% of the population live in poverty, and of those 92% live in rural areas [8]. The official language is French. The Health and Demographic Surveillance System (HDSS) site of the Nouna Health Research Centre (CRSN, Centre de recherché en santé de Nouna) is located in the north-west of Burkina Faso in the province of Kossi, 300 km from the capital Ouagadougou. In 2008, the Nouna HDSS counted approximately 81,500 inhabitants of various ethnic groups, in an area covering 1,756 km^2^. The principal occupation is subsistence farming. This Sahelian dry tropical Savannah region sees annual wet seasons from around June to October, during and directly after which time physician-diagnosed malaria increases dramatically [9]. Recently published childhood mortality rates still show a high physician-assessed burden of malaria, which has only slightly decreased over the last few years, if at all [10,11].

General results of the comparison of PCVA and the current third version of InterVA (InterVA-3) for this population using an INDEPTH VA questionnaire were recently published [12]. Both the model (63.1%) and physicians (70.8%) determined infectious diseases as causing a larger proportion of deaths than chronic disease. However, substantial differences were observed in terms of the proportion attributed to each disease as cause of death by the two systems. While physicians determined malaria (31.4%), diarrhoea (10.1%), pneumonia/sepsis (7.9%) and cardio-vascular diseases (5.1%) as the most important causes of death, the model determined diarrhoea (15.0%), meningitis (12.3%), pneumonia/sepsis (11.7%), malaria (11.1%) and tuberculosis (6.5%) as the most important causes [12].

The reasons for these sizeable differences in cause of death diagnosis, especially malaria, are unclear. Diagnosing malaria based on symptoms, without laboratory confirmation, is difficult and poses a particular challenge for VA. One hypothesis for the discrepancy between the model's and physicians' malaria diagnoses could be that local physicians had more information on which to base their diagnosis than did the model. Both systems of diagnosis used information obtained from the locally designed verbal autopsy questionnaire, but the model used only information from structured items linked to indicators informing the model, while physicians also read a narrative or "free-text" section right at the beginning of the questionnaire. Not all information in the questionnaire could be linked to InterVA-3 indicators informing the model, and not all indicators had matching information in the questionnaire. This mismatch contributed to 37 of 106 total possible indicators being left blank, though 14 of these indicators require a clinical diagnosis, difficult to obtain for most of the population in the Nouna region [12]. As the free-text is not standardised and therefore not available electronically, it did not feed into the model. It was hypothesised that doctors may be influenced by information in this free-text and thus could differ from the model in their determination of cause of death. Free-texts might have provided an opportunity to describe conditions not captured in the structured questions, leading to more malaria diagnoses. In the Nouna INDEPTH site, the free-text section of the verbal autopsy questionnaire is consistently filled out and generally contains a chronological description of symptoms, medications taken (both modern and traditional), and the development of circumstances leading up to death. Physicians likely paid particular attention to the free-texts, rendering their inclusion in the model an important consideration.

There has been debate about the impact of free-text on verbal autopsy cause of death determination. Fottrell found in a maternal mortality study in the same Burkina Faso site that including information from free-texts did not add significant information to influence the model's outcome by more than 1% (using InterVA-M for women of reproductive age) [13]. Inclusion of free-text was also found to be negligible in the multi-site Population Health Metrics Research Consortium study using a questionnaire based on the World Health Organization (WHO) standardised VA questionnaire [14]. In contrast, Marsh *et al *found that a combination of free-text and structured questionnaire produced neonatal cause of death diagnosis in Pakistan most closely aligned with clinical diagnosis, as compared with a combination of only structured questionnaire and computer modelling [15]. Gajalakshmi and Peto also found in a study in North India that use of narrative sections of the questionnaire in conjunction with structured sections more accurately diagnosed causes of death than use of structured sections alone; in fact narrative texts alone were also found to be more informative than use of structured sections alone [16].

Thus, we decided to include information from comprehensive free-text in order to determine whether and how InterVA-3 cause of death outcomes in this malaria holo-endemic region were influenced, and whether the gap between the doctors' and the model's cause of death determination would be narrowed.

## Methods

### Free-text processing

Free-text information was extracted from three sub-groups of the overall sample: a representative sub-sample and two samples based on malaria diagnosis by physicians and the model, diagnosed with malaria by one system but not the other. All electronically available VA questionnaires (N = 5649) of the Nouna HDSS for the years 1998 to 2007 build the source for the underlying free-text analyses, and only those records with electronic scans were included.

**Figure 1:**
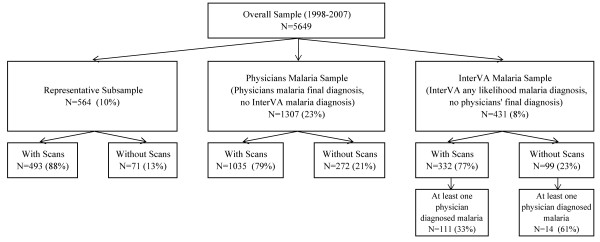
**Construction of sub-groups for free-text analyses**.

As displayed in Figure 1, the first sub-group ("representative sample") was a randomly drawn sample of 10% of the overall sample (N = 564, of whom 493 had scans available), taking into account the total age and sex distribution.

The second sub-group ("physicians malaria sub-group") included all individuals for whom the final cause of death by doctors was determined to be malaria and for whom the model did not determine malaria as either the first, second or third likely cause of death (N = 1,307, of whom 1,035 had scans available). Individuals who were diagnosed with malaria by only one physician were not included in this sub-group, if the final diagnosis was another disease or indeterminate.

The third sub-group ("InterVA-3 malaria sub-group") included all individuals for whom the InterVA system determined malaria as either the first, second or third likely cause of death, and for whom the final cause of death by doctors was not determined to be malaria (N = 431, of whom 332 had scans available). This latter group includes those individuals for whom one doctor determined malaria to be the cause of death, but was overruled by two other doctors in the final cause (N = 125, of whom 111 had scans available).

The first step to including free-text information in the model required summarising all free-texts in such a way that they could be allocated to indicators. Any symptoms described in the free-text were summarised in standardised fashion, separated electronically into individual symptoms, and, wherever possible, matched to one of the 106 indicators available in the InterVA programme.

These data were then merged with the original dataset in which indicators had been set according to information from the standardized section of the Nouna questionnaire. If indicators were set to "yes" according to information from free-text but were not set to "yes" according to information from the standardised section, the indicator was changed to "yes". However, free-texts are assumed to provide additional but not all information about an individual's symptoms. Therefore, any indicators that had previously been set to "yes" according to information from the standardised section were not changed, even if no information was available on a given indicator in the free-text. Once all indicators were modified according to additional free-text information, the model was then re-run with modified indicator information according to free-texts.

Ethical approval for this study was granted by the ethical committee of the University of Heidelberg and by the local ethical committee of the Centre de Recherche en Santé de Nouna.

### Data analysis

Cause-specific mortality fractions (CSMFs) for individual diagnoses before/after free-text adaptation were compared using absolute differences in all three sub-groups. The weighted InterVA and individual physician CODs were compared by determining agreement in each category. For example, if a COD counted as 15% in PCVA and 10% in InterVA, the minimum of the two (i.e. 10%) was regarded as the agreement at the population level, regardless of whether the same records were included by physicians and the InterVA in that 10%or not. The sum of the minima for all CODs was considered the "concordance" of the two methods [12], and such overall concordances between models including and excluding free-text were compared to measure degree of free-text's improvement of models. Data preparation and some analyses were conducted in Stata 10. Some data analysis was conducted in SAS 9.

## Results

### Population characteristics

As described in Table 1, the overall distribution of the sample showed a large concentration of deaths among the young (45.4% under age five) and the old (23.6% aged 64 and above). Two thirds of deaths occurred during the dry season. These age and sex distributions were also reflected in the representative sample, with a slightly higher percentage of deaths in the dry season.

**Table 1:** Population characteristics of the overall sample and sub-groups

		All death records 1998-2007			Sub-groups		
Variable		Total Sample %	Malaria by Physicians^1 ^%	Malaria by InterVA3^2 ^%	Representative Sample %	Physicians Malaria Sub-group^3 ^%	Model Malaria Sub-group^4 ^%
N		5649	1804	954	493	1035	332
Age	<4 weeks	3.9	1.3	0.0	3.9	2.1	0.0
	4 weeks-1 year	15.0	27.2	24.7	14.2	27.3	18.1
	1-4 years	26.5	44.0	40.6	25.8	42.4	25.3
	5-14 years	6.1	5.9	5.8	5.9	5.6	7.2
	15-49 years, male	7.6	2.6	6.2	7.5	2.1	11.1
	15-49 years, female	7.6	2.1	12.7	8.3	1.0	26.5
	50-64 years	9.8	2.9	3.1	10.5	3.4	4.5
	65+ years	23.6	14.0	6.9	23.9	16.0	7.2
	Mean	29.5	15.0	13.9	30.0	16.6	20.9
Sex	Male	51.6	50.1	47.6	51.9	48.	41.0
	Female	48.4	49.9	52.4	48.1	52.0	59.0
Season	Wet	39.7	48.7	59.3	34.7	41.8	53.3
	Dry	60.3	51.3	40.7	65.3	58.2	46.7

^1 ^All Cases with final physicians malaria diagnosis, including overlap with InterVA malaria diagnoses

^2 ^All Cases with any likelihood malaria diagnosis. by InterVA, including overlap with physicians' malaria diagnoses

^3 ^Cases in which physicians diagnosed malaria as final diagnosis and excluding InterVA-3 malaria diagnoses of any likelihood

^4 ^Cases in which InterVA-3 diagnosed malaria with any likelihood and excluding physicians' final malaria diagnoses

In contrast, the two malaria sub-groups differed in varying respects from the overall sample's distribution. These two sub-groups do not represent all malaria diagnoses by doctors and the model; therefore, Table 1 additionally shows the distributions of all doctors' final and any-likelihood model malaria diagnoses in order to highlight particularities to the sub-groups. Overall, both the model and physicians diagnosed most malaria in children under five years of age. The proportion of malaria diagnoses by physicians aged 65 and above was twice as high (14.0%) as amongst those diagnosed with malaria with any likelihood by the model (6.9%). Amongst those diagnosed as malaria with any likelihood by the model, 12.7% were women of reproductive age, while amongst physicians' final diagnoses that proportion was only 2.1%. Hence, slightly more females overall were diagnosed with malaria by the model than were males, while the proportions male and female were the same amongst physicians' final diagnoses.

### Free-texts in the representative sub-group

**Figure 2:**
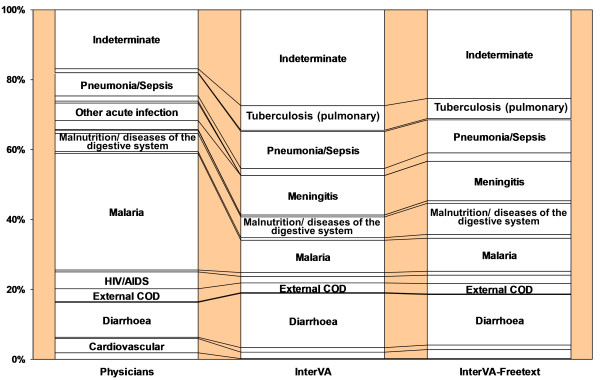
**Physicians' and InterVA diagnoses in the representative sample: before and after free-text incorporation**.

Table 2 lists the concordances between physician and various model diagnoses. When describing differences in model output, we refer to 100 minus the concordance between two models. Incorporating free-text in the representative sample resulted in a 5.5% such difference in model output (see Figure 2), but without changing the overall pattern of CODs. It should be noted, however, that comparing overall proportions between two models does not reveal those instances in which cases replace one another at the individual level, rendering changes at the population level undetectable. The discordance between the models before and after free-text incorporation is relatively evenly distributed across the disease categories, with the exception of a larger increase in malnutrition/diseases of the digestive system. The malaria proportion remained the same.

**Table 2:** Overall Concordances between various models and physicians (all disease categories)

Model Type	Comparison	Representative Sample (%)	Physicians Malaria Sub-group (%)	Model Malaria Sub-group (%)
Original InterVA	with physicians	62.1	0.0	36.3
Free-text	with physicians	64.0	2.7	46.4
	with original InterVA	94.5	94.5	89.8

### Free-texts in sub-groups with discrepancies in malaria diagnoses

**Figure 3:**
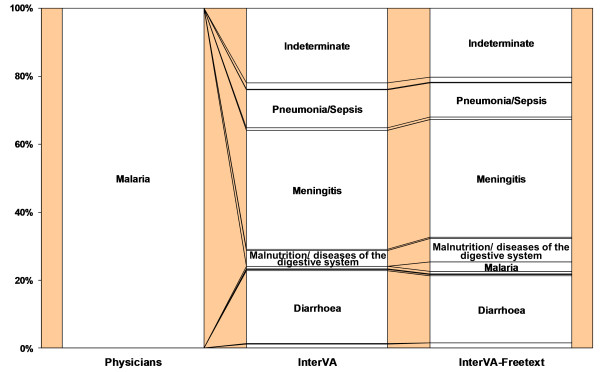
**Physicians' and InterVA diagnoses in the physicians malaria sub-group: before and after free-text incorporation**.

In the physicians malaria sub-group, there was a 5.5% absolute difference between the models before and after incorporation of free-text (see Table 2). Changes in individual disease categories were generally between 0 and 3%, with a smaller than expected increase in malaria (from 0% to only 2.7%) (see Figure 3).

**Figure 4:**
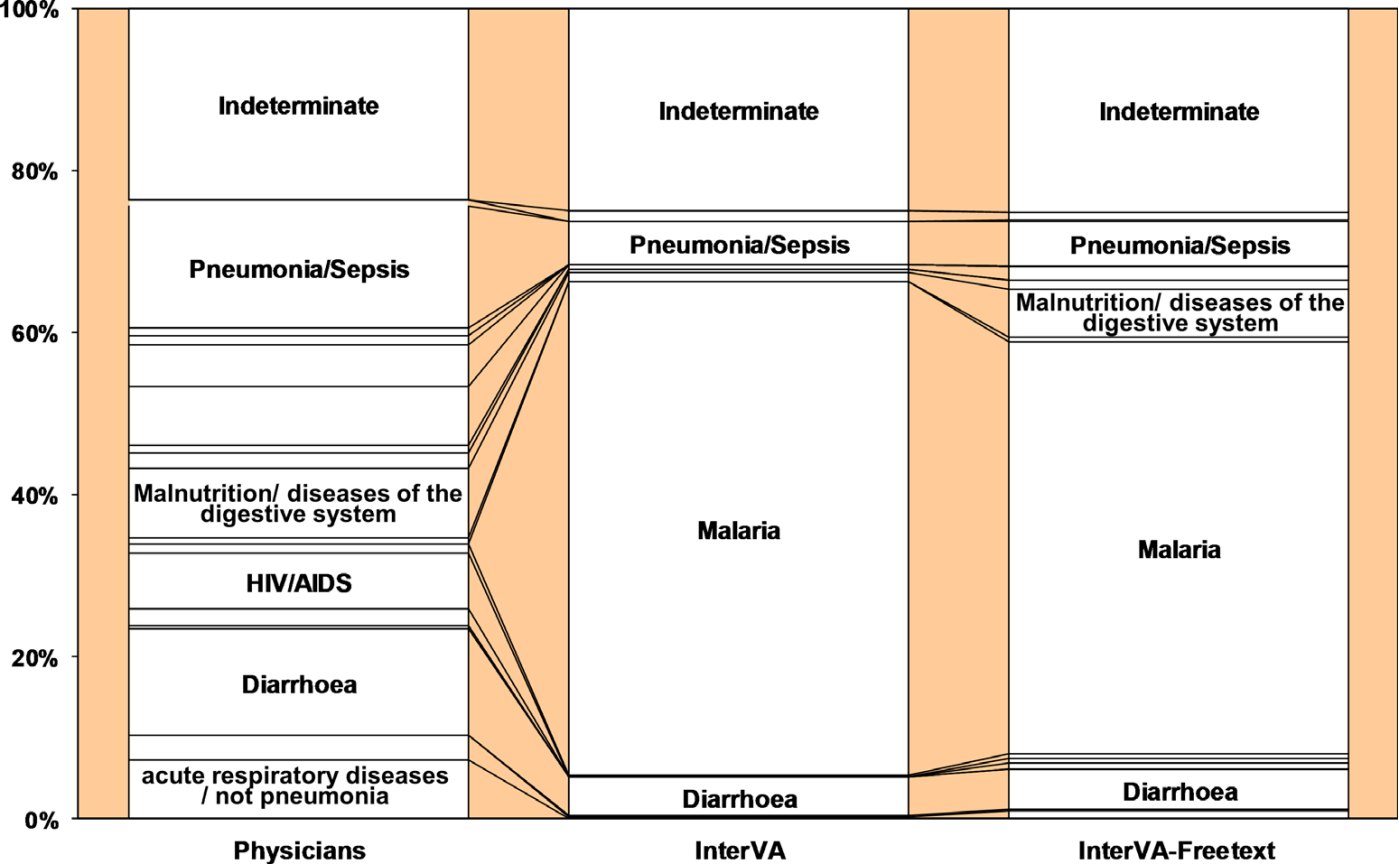
**Physicians' and InterVA diagnoses in the model malaria sub-group: before and after free-text incorporation**.

The greatest change in model output due to free-text incorporation was seen in the model malaria sub-group (10.2%) (see Table 2), with most of the absolute difference between the models accounted for by a 9.9% decrease in malaria diagnosis. There is no other diagnosis which saw a comparably dramatic increase (see Figure 4).

Overall, the diagnosis which consistently showed increases through incorporation of free-text in all three sub-groups was malnutrition/diseases of the digestive system, with increases in diagnosis of such diseases of 2.9% in the representative sample, 2.3% in the physicians malaria sub-group, and 4.6% in the model malaria sub-group.

Visible, particularly in the two malaria discrepancy sub-groups, is the co-dependency of malaria and pneumonia/sepsis, diarrhoea, and meningitis. In the physicians malaria sub-group, while malaria increased, pneumonia/sepsis, diarrhoea, and meningitis decreased. The opposite was true in the model malaria sub-group. In the representative sample, no population-level changes were noted in malaria and meningitis, though pneumonia/sepsis and diarrhoea both saw decreases.

Indeterminate causes decreased by 2.1% in the representative sample and by 1.6% in the physicians malaria sub-group, though there was no notable change in the model malaria sub-group. The representative sample also saw a slight increase in the CSMF for cardiovascular disease and a reduction in tuberculosis.

### Effect of free-texts, in various age groups

The impact of free-texts varied by age: across all three sub-groups, the greatest impact was seen amongst children aged 5-14 years, while the smallest impact was seen amongst children under the age of five and those aged 65 and above.

Details of changes by age are not shown, but changes of greater than 3% in the representative sample include (1) amongst neonates: a 4.4% increase in perinatal disease and 3.3% decrease in indeterminate causes; (2) amongst under fives: a 3.6% increase in malnutrition and diseases of the digestive system; (3) amongst children 5-14: a 4.1% increase in cardiovascular disease and 12.1% in malnutrition/diseases of the digestive system; and a 4.5% decrease in malaria and 3.7% in meningitis; (4) amongst female adults: a 4.0% decrease in indeterminates; (5) amongst male adults: a 6.0% increase in malnutrition/diseases of the digestive system and 7.6% decrease in tuberculosis; and (6) amongst those aged 50-64: a 3.2% increase in cardiovascular disease.

### Malaria in free-texts, in various age groups

Though malaria diagnoses in the physicians malaria sub-group increased in all age groups, increases were more pronounced amongst those aged younger than 50, ranging from 2.7% to 4.6%, with the exception of no change amongst neonates. In the model malaria sub-group, decreases in malaria diagnoses were minimal amongst those younger than one year, but ranged from decreases of 6.3% to 18.4% in all remaining age groups.

With respect to malaria, the representative sample saw both decreases and increases, after incorporating free-text, with a decrease of 4.5% amongst children aged 5-14 and changes of less than 2% in all other age groups.

### Effect of free-texts, according to season

The representative sample saw a greater impact of free-text for those who died in the dry season (7.1% change in the model output), as compared to those who died in the wet season (3.7% change). Only marginal differences were observed in the other two sub-groups comparing changes in the wet versus dry seasons (physicians malaria sub-group: 5.9% versus 5.3% change, model malaria sub-group: 10.1% versus 10.9% change).

### Changes in numbers of indicators set through free-text

On average, individuals in all three sub-groups gained only a third to half of one indicator through the addition of free-text information, a pattern that did not vary by age (data not shown). The maximum number of indicators additionally set for any individual was four, in all three sub-groups. No age group in any sub-group saw no individuals gain any indicators through free-text.

Table 3 outlines indicators for which information was gained through free-text by sub-group. Overall, information on 13/106 indicators appeared at least once in the free-texts that had not been matched to any item in the structured, electronically available portion of the Nouna questionnaire: abdominal mass, anaemia, drowning, excessive urination, fall, heart disease, liver disease, oral candidiasis, poisoning, surgery, swelling of breasts, swelling of glands, and jaundice (see Table 3). Ten new indicators appeared in the representative sample, five in the physicians malaria sub-group, and 11 in the model malaria sub-group. Indicators which saw an additional information increase for at least 1% of each sub-group were abdominal swelling, jaundice, any swelling of ankles or legs, any skin lesions or ulcers, and anaemia or paleness. The physicians malaria sub-group and representative sample also saw the addition of information on heart disease for over 1% of the sub-group.

**Table 3:** Indicator information added through free-text

	Representative Sample N = 493		Physician Malaria Sub-group N = 1035		Model Malaria Sub-group N = 332	
	counts without free-text	added counts^1^	counts without free-text	added counts^1^	counts without free-text	added counts^1^
	N	N (%)	N	N (%)	N	N (%)
was death very sudden or unexpected	81	4 (0.8)	204	12 (1.2)	10	2 (0.6)
did s/he drown	0	5 (1.0)	.		.	
any poisoning, bite, sting	0	6 (1.2)	0	3 (0.3)	0	8 (2.4)
any headache	86	8 (1.6)	121	21 (2.0)	101	6 (1.8)
any stiff neck	29	1 (0.2)	207	4 (0.4)	13	6 (1.8)
any rigidity/lockjaw	77	4 (0.8)	394	15 (1.4)	43	2 (0.6)
any chest pain	86	3 (0.6)	72	5 (0.5)	53	4 (1.2)
any abdominal pain	105	7 (1.4)	117	25 (2.4)	91	7 (2.1)
any diarrhoea with blood	102	9 (1.8)	222	8 (0.8)	53	4 (1.2)
any abdominal swelling	0	27 (5.5)	0	45 (4.3)	0	38 (11.4)
any yellowness/jaundice	0	27 (5.5)	0	80 (7.7)	0	14 (4.2)
any urinary retention	11	5 (1.0)	14	1 (0.1)	6	3 (0.9)
any swelling of ankles/legs	9	25 (5.1)	8	30 (2.9)	3	29 (8.7)
any skin lesions or ulcers	43	32 (6.5)	42	39 (3.8)	29	33 (9.9)
any rash (non-measles)	23	1 (0.2)	20	1 (0.1)	21	4 (1.2)
any acute fever	215	2 (0.4)	755	13 (1.3)	.	
any facial swelling	4	8 (1.6)	1	8 (0.8)	2	7 (2.1)
any anaemia/ paleness	0	13 (2.6)	0	51 (4.9)	0	11 (3.3)
any drowsiness	268	9 (1.8)	526	14 (1.4)	220	6 (1.8)
any diagnosis of heart disease	0	12 (2.4)	0	9 (0.9)	0	19 (5.7)
any surgery just before death	7 (1.4)	6 (1.2)	0	1 (0.1)	0	3 (0.9)

^1^added counts = additional number of observations for which indicators were set after incorporation of free-text

All indicators which changed between 0 and 1%: was she pregnant at death; did pregnancy end < 6 weeks; did final illness last < 3 weeks; did final illness last at least 3 weeks; had s/he fallen recently; any obvious recent injury; any convulsions or fits; any diagnosis of epilepsy; was the fontanelle raised; was the fontanelle sunken; paralysis on both sides; any chest indrawing; cough for up to 3 weeks; cough for > 3 weeks; any rapid breathing; any difficulty breathing; any breast lump or lesion; any wheezing; any abdominal mass; any acute diarrhoea; any vomiting; any abnormality of urine; any haematuria; no bilateral swelling of ankle; any measles rash; any excessive water intake; any excessive urination; any persistent fever; any enlarged/swollen glands; was there a comma > 24 hours; any weight loss; any diagnosis of hypertension; any diagnosis of liver disease

## Discussion

The aim of this study was to assess whether physicians' access to free-texts explains the large discrepancy with the InterVA-3 model in malaria diagnoses in this holo-endemic malaria region, not to judge whether one method or the other is superior in its accuracy of malaria diagnosis. Overall we found that access to free-text does not explain why physicians diagnose more malaria than does the model. Nonetheless, information from free-text was found to provide additional information not available in the structured portion of the questionnaire, which resulted in non-negligible changes in the model output.

Not surprisingly, the highest proportion of malaria diagnoses by both the physicians and the model was amongst under five year olds and during the wet season. However, both methods diagnosed malaria in some age and sex groups in ways contradicting the standard knowledge on malaria. Physicians diagnosed malaria within the first four weeks of life, generally accepted not to be possible [17], while such diagnoses did not occur in model output. Also, it was notable that amongst physicians' malaria diagnoses there was a higher proportion aged 65 and above, and amongst the model diagnoses there was a higher proportion of women of reproductive age. Very little literature exists on malaria deaths amongst the elderly, likely due to lower sample sizes in this age category and other causes of death with greater priority. However, perhaps physicians are justified in diagnosing more malaria amongst the elderly, whose weakened immune systems may be less able to combat the disease than those aged below 65. A review of malaria mortality rates in sub-Saharan Africa and Bangladesh found that malaria death rates drop in late childhood and young adulthood, and then steadily increase with age in West and East Africa [18]. A study in India also suggests malaria mortality rises in older ages, though the epidemiology of malaria mortality may be different in India as compared to Burkina Faso [19]. This trend was not present in the "Global Burden of Diseases and Risk Factors" report, which showed consistently decreasing malaria rates with increasing age, for sub-Saharan Africa as a whole [20].

The model estimates higher levels of mortality due to malaria amongst women of reproductive age than physicians. Findings by Lemma *et al *suggest that women of reproductive age are more likely to die of malaria compared with other adults, supporting the trends indicated in malaria mortality in model output [21].

Although the InterVA has recently been validated with varying types of clinical data, it remains difficult to judge whether physicians or the model may be closer to the real causes of death, as contradicting results were seen under different conditions [14,22,23]. Unfortunately, this study suffers from a lack of a gold standard, as do all verbal autopsy-related studies based in developing country communities without full access to health services, and thus the true causes of death are impossible to be obtained [24]. This problem is particularly challenging for malaria, a disease that is difficult to diagnose accurately without parasitic evidence, and from which fatality is much reduced when health services are accessed, rendering in-person validation by a physician near impossible [19,25-27].

Had the free-text hypothesis been correct, one would have expected a closing of the gap in malaria diagnoses between physicians and the model. Though absolute changes between models were small in both malaria-specific sub-groups, trends tended toward an increase in model malaria diagnosis in the physicians malaria sub-group and a decrease in the model malaria sub-group. This trend was not evident when comparing absolute differences in proportions at population level for the representative sample, though separate analyses by age group revealed increases in malaria diagnoses in the youngest and oldest age groups, but a decrease in 5-49 year olds. However, additional shifts in malaria diagnoses at the individual level may have cancelled each other out at the population level. A more important closing of the malaria diagnosis gap was achieved when setting the model malaria indicator to "yes" when any fever was present, without regard to duration or treatment [12]. This suggests that physicians are highly influenced by presence of any fever when diagnosing malaria, but also shows the sensitivity of the model to the malaria indicator being set to "yes", despite difficulties in obtaining clinical diagnosis of this disease in developing countries.

The most consistent outcome in all three sub-groups in the free-text analysis was a rise in malnutrition/diseases of the digestive system. This is a result of the availability of information on jaundice (a principal symptom of liver disease) in the free-text, an item not captured in the questionnaire. In a further sub-group analysis of the effects of free-text on HIV diagnosis, the sample was too small to see any HIV-related impact, but even in the smaller sub-group, the greatest change was an increase in malnutrition/diseases of the digestive system (data not shown).

A notable pattern was visible in the interplay of three disease categories with similar symptoms to malaria: pneumonia/sepsis, diarrhoea, and meningitis. These infectious diseases all display fever, some cough, and some convulsions (in severe cases [17,28]). While physicians may struggle to distinguish between these causes without having seen the individual, obtaining laboratory tests, or, in many cases, having full information on the circumstances of death, the model also shows inter-dependent fluctuations, which are visibly sensitive to additional free-text information. In particular, meningitis and malaria appear to have overlapped in diagnosis, the model diagnosing about twice as much meningitis as physicians did in the original model. Indeed, Burkina Faso is one of the core countries of the meningitis belt, where more than 90% of all meningitis cases occur during calendar weeks 1-20, corresponding to the meningitis season, with the typical bell shaped incidence curve peaking by end of March and seen every year at district level [29]. As malaria is endemic throughout the year, during the meningitis season both diseases are present, with the potential for misdiagnosis of the one or the other. In some individuals a bacterial infection and malaria parasitaemia may even occur simultaneously (as seen in children) [30,31]. The model's season indicator could thus result in too few malaria diagnoses in the dry season.

Though the over-all change in the model outputs was not monumental, consulting the free-text did reduce the number of unallocatable indicators from 37 to 24, in the three sub-groups combined. Additional free-text information seemed to be evenly distributed amongst all age groups, without regard to type of sub-group, indicating no particular benefit of free-text inclusion for any particular sub-population.

Ideally the VA questionnaire used would have been tailored precisely to the indicators such that there would be no unallocatable indicators. However, if a questionnaire does not have information for certain indicators, this analysis shows that it is possible to search in the free-texts for information on missing indicators (assuming free-texts of reliable consistency). A reduction in indeterminate causes also indicates fuller information as a result of the free-text information. The Nouna INDEPTH site is in the process of switching to an improved questionnaire, universally employed across the INDEPTH sites, and hence consulting free-text may have less of an impact for this site. However, with a plethora of VA questionnaires still in use in developing countries, it is undoubtedly useful to observe the impact free-text may have when searching for information not gathered in the structured portion of the questionnaire. Consulting free-text may be especially relevant for studies using historical data, in which data may have been gathered in the free-text not known at the time to be relevant for the structured portion. Thus, even if electronic coding should become the norm for VA analysis, free-texts should be maintained as part of VA questionnaires and interviewers trained on documenting narratives in a consistent manner.

## Conclusions

Free-texts do not explain discrepancy in malaria diagnoses between PCVA and InterVA-3, though they are useful to consult for those indicators that cannot be set using structured portions of the questionnaire. Such an effect is likely to be seen for any electronic VA analysis method based on inputted information from structured questionnaires. Though free-text analysis is a time-consuming process, software such as READ-ME, described by Murray *et al*, may render their incorporation more feasible [24]. Free-texts may provide useful information in cases where questionnaires are not fully comprehensive or for retrospective studies measuring items currently not measured.

## Competing interests

The authors declare that they have no competing interests.

## Authors' contributions

JR drafted the paper, performed statistical analyses, and contributed to the allocation of the model requirements. EL performed statistical analyses and contributed to the allocation of the model requirements and writing of the paper. FN, MY and AS participated in the conception, in particular with regard to the Nouna VA data, and contributed to the writing of the paper. HB contributed to the writing of the paper. HR designed and supervised the study, contributed substantially to the writing of the paper, and supervised statistical analyses. All authors read and approved the final version of the manuscript.
